# Workplace violence and depressive symptoms: the mediating role of fear of future workplace violence and burnout among Chinese nurses

**DOI:** 10.1186/s12888-024-05827-8

**Published:** 2024-05-21

**Authors:** Chaofan Li, Chang Fu

**Affiliations:** 1 Centre for Health Management and Policy Research, School of Public Health, Cheeloo College of Medicine, Shandong University, No. 44 Wenhua Xilu Road, Jinan, Shandong China; 2 NHC Key Lab of Health Economics and Policy Research, Shandong University, No. 44 Wenhua Xilu Road, Jinan, Shandong China; 3 Department of Health Service and Management, School of Health Management, Binzhou Medical University, No. 346 Guanhai Road, Yantai, Shandong 264003 China; 4 Department of Health Psychology, School of Nursing and Rehabilitation, Cheeloo College of Medicine, Shandong University, No. 44 Wenhua Xilu Road, Jinan, Shandong 250012 China

**Keywords:** Workplace violence, Depressive symptoms, Fear of future workplace violence, Burnout, Nurse

## Abstract

**Background:**

The mechanisms underlying the relationship between workplace violence (WPV) and depressive symptoms in nurses have been less studied. This study aims to examine the mediating role of fear of future workplace violence (FFWV) and burnout in the association between WPV and depressive symptoms.

**Methods:**

We conducted a cross-sectional web survey at 12 tertiary hospitals in Shandong province, China, in 2020. The Center for Epidemiologic Studies Depression Scale (CESD-10), the Chinese version of the Maslach Burnout Inventory-General Survey and the Fear of Future Violence at Work Scale were used to collect data. Descriptive statistics, independent sample t-test, one-way analysis of variance, Pearson’s correlation coefficient, and ordinary least squares regression with bootstrap resampling were used to analyze the data.

**Results:**

The prevalence of depressive symptoms was 45.9% among nurses. The regression model showed that FFWV and burnout mediated the relationship between WPV and depressive symptoms. The total effects of WPV on depressive symptoms (3.109, 95% bootstrap CI:2.324 − 3.713) could be decomposed into direct (2.250, 95% bootstrap CI:1.583 − 2.917) and indirect effects (0.769, 95% bootstrap CI:0.543 − 1.012). Indirect effects mediated by FFWV and burnout were 0.203 (95% bootstrap CI:0.090 − 0.348) and 0.443 (95% bootstrap CI:0.262 − 0.642), respectively. Furthermore, serial multiple mediation analyses indicated that the indirect effect mediated by FFWV and burnout in a sequential manner was 0.123 (95% bootstrap CI:0.070 − 0.189).

**Conclusion:**

The prevalence of depressive symptoms among Chinese nurses was high. The WPV was an important risk factor for depressive symptoms and its negative effect was mediated by FFWV and burnout. The importance of decreasing WPV exposure and level of FFWV and burnout was emphasized to prevent depressive symptoms among nurses. The findings implied that hospital managers and health policy makers should not only develop targeted interventions to reduce exposure to WPV in daily work among all nurses, but also provide psychological support to nurses with WPV experience to reduce FFWV and burnout.

## Introduction

Nurses play an irreplaceable role in assisting in diagnosing and treating patients, saving lives, alleviating pain, and promoting the rehabilitation of patients. Due to long work hours and high work pressure, nurses’ health is very likely to be negatively affected by depression [1]. Depression often involves a loss of interest in activities that were once enjoyable, feelings of worthlessness or guilt, difficulty sleeping, changes in appetite, and decreased energy or motivation [2], and the high prevalence of depressive symptoms among nurses has become a global problem [3]. Previous studies found that the prevalence of depressive symptoms among American nurses was 18% [4], while that among Chinese nurses was significantly higher, 48.83% [5]. Depressive symptoms not only affect nurses’ health and well-being, but also negatively impact work quality, for example, increased medical errors and reduced patient satisfaction [1]. Therefore, it is urgent and vital to reduce the occurrence of depressive symptoms among nurses.

Compared to other professions, nursing is one of the most exposed occupations to workplace violence (WPV) [6], which is a serious issue worldwide [7]. In recent years, WPV experienced by nurses has become increasingly common, drawing the attention of researchers worldwide. WPV refers to violence experienced by people on the job or on duty [8]. The WPV suffered by nurses has been classified into verbal, psychological, and physical violence [9]. A study by Kobayashi et al. found that 44% of Japanese nurses had experienced WPV in the past year [10], while in the United States, 43% of nurses had experienced WPV in the same time frame [11]. Furthermore, a review showed that 71% of nurses in China had experienced WPV in the past 12 months [12]. Several studies have reported that WPV can be associated with the development of depressive symptoms [13–15]. In recent years, due to the high incidence rate of WPV experienced by nurses worldwide, the mechanism of how WPV affects nurses’ health outcomes has become the key focus of researchers in this field. Previous studies have shown that WPV not only has direct effects on nurses’ health outcomes, but also indirect effects [16–18]. Therefore, the relationship between WPV and nurses’ health outcomes is likely to be highly complex. However, few studies have explored the potential mechanisms underlying the relationship between WPV and depressive symptoms in nurses.

Fear of future workplace violence (FFWV) is an important outcome of WPV and is defined as an emotional response to an individual’s risk of victimization of WPV [19]. Nurses who have experienced WPV can develop high levels of FFWV [20]. Studies in western countries have shown that FFWV is associated with negative outcomes in individual work performance and health, such as turnover intention [21] and burnout [22]. In addition, Barling reported that FFWV can mediate the effects of WPV on personal outcomes [23]. Therefore, in this study, we hypothesized that FFWV is a potential mediating variable between WPV and depressive symptoms in nurses.

Nursing is one of the occupations that show a strong tendency to burnout [24]. Burnout refers to the state of physical and mental fatigue and exhaustion experienced by individuals under pressure of work, and is officially recognized as a serious health problem by the World Health Organization [24]. Burnout has three dimensions: emotional exhaustion, cynicism, and decreased personal accomplishment [25]. A previous study found a significant association between burnout and depressive symptoms among nurses [26]. Another study reported that WPV may contribute to burnout among nurses and physicians [27]. Furthermore, Havaei indicated that burnout mediated the relationship between WPV and health outcomes of individuals, such as musculoskeletal injuries and anxiety disorders [17]. Therefore, we hypothesize that burnout may be a potential mediating variable between WPV and depressive symptoms. Previous studies have suggested that WPV has a positive impact on FFWV [20], and that FFWV is associated with a high level of burnout among nurses [22]. In contrast, Rogers and Kelloway indicated that violence affects organizational outcomes (such as emotional attachment and turnover intention) via FFWV [28]. Based on previous studies, we hypothesized that the experience of WPV is associated with an increase in FFWV and consequently increased burnout, which in turn is related to an increase in the prevalence of depressive symptoms.

Although previous studies have examined several mediating variables between WPV and depressive symptoms, few studies have investigated the mediating roles of FFWV and burnout in the relationship between WPV and depressive symptoms among nurses. Examining the potential mediating variables between WPV and depressive symptoms of nurses will help unravel the underlying relationships and provide useful information to policy makers and hospital administrators for the development of effective interventions to improve their mental health.

Therefore, this study aimed to: (1) investigate the prevalence of WPV, FFWV, burnout, and depressive symptoms among Chinese nurses; (2) explore the degree to which there is a direct association between WPV and depressive symptoms; and (3) examine the mediating roles of FFWV and burnout in the relationship between WPV and depressive symptoms.

## Methods

### Study design and participant recruitment

Data were obtained through a cross-sectional web survey conducted in Shandong province, China, between July and September 2020. In the present survey, a multistage random sampling method was applied. First, 16 perfecture-level cities in Shandong province were classified into three categories (high, medium, and low) based on the per capita GDP level. Two cities were randomly chosen from each category. Second, two tertiary hospitals were randomly selected in each city. Third, two thirds of the departments including internal medicine, surgery, obstetrics and gynecology, pediatrics, and emergency were selected from each hospital sample. All nurses in selected departments were invited to participate in the survey [20]. The inclusion criteria were those who were registered nurses, employed by the hospital, and willing to participate in this survey. Exclusion criteria were those who were on vacation or temporary leave for their continuing education, or had serious mental disease other than depression or physical disorders that may hinder their participation.

In this study, a single population proportion formula was used to calculate the minimum sample size, n = Z_α/2_^2^p(1-p)/d^2^ [20]. Based on the assumption: the prevalence of depressive symptoms among nurses in China was 31.12% [5], marginal error of 5%, 95% confidence interval (CI) (α = 0.05), *n* = 1.96^2^ × 0.3112(1-0.3112)/0.05^2^ = 329.39 ≈ 330. Considering a design effect of 2 (multistage sampling technique) [29] and adding a non-response rate of 20% in this study, the final sample size was 729 nurses [330 × 2 × (1 + 0.2)].

The electronic questionnaire was created applying ‘Wenjuanwang’ (https://www.wenjuan.com/*).* The web page of the questionnaire was sent to participants’ mobile phones using *WeChat* (a social media app). A total of 1,933 nurses from 12 tertiary hospitals participated in this survey and 40 participants did not finish the questionnaire. Due to the low proportion of incomplete questionnaire (2.1%), after excluding cases with missing data, 1,893 cases were finally included in the analysis. The response rate was 97.93%.

### Ethical approval and consent to participate

The research was approved by the Ethical Review Committee of the School of Nursing and Rehabilitation of Shandong University (approval number: 2020-R-50). All subjects gave their informed consent for inclusion before participating in the survey. All the methods in the present study were carried out in accordance with the relevant guidelines and regulations.

### Measurement

#### Depressive symptoms

The main dependent variable was depressive symptoms, which were measured using a widely used and freely available questionnaire, the 10-item Center for Epidemiologic Studies-Depression (CESD-10) scale [30]. The CESD-10 consists of 10 self-reported items that assess each symptom experienced during the past week, using a four-point Likert scale ranging from 0 (rarely or none ) to 3 (all the time). The total score is the sum of all 10 items, ranging from 0 to 30. Higher scores represent more severe depressive symptoms. A previous study demonstrated that the Chinese version of CESD-10 has high reliability and validity [31]. The Cronbach’s α of CESD-10 in this study was 0.68 which showed that the reliability was minimally acceptable.

#### Workplace violence

This was measured by asking respondents ‘Whether you have experienced verbal, psychological, or physical violence at your workplace from patients, and/or their relatives, your colleagues and/or leaders during the past years?’ [32]. Verbal violence included abuse, sarcasm, indignity, etc. Psychological violence included baseless charges or complaints, slander, attempts to damage reputation, destruction of public facilities, booing, maliciously taking pictures, glowering, sexual harassment, etc. Physical violence included biting, pushing, fighting, cutting, sexual assaults, etc [20]. If the respondent answered ‘Yes’, it was coded as 1, and ‘No’ was coded as 0.

#### Fear of future violence at work

The magnitude of fear of experiencing violence at work within the next year was measured using a modified version of the inventory developed by Schat and Kelloway [33]. The modified version of the inventory was made up not only the 10 items proposed by Rogers and Kelloway [28], but also 2 items used by Barling et al. [34]. Each item was rated on a seven-point Likert scale ranging from 1 (strongly disagree) to 7 (strongly agree). The total score was the sum of the 12 items, with high scores indicating a high degree of FFWV. This inventory was translated into Chinese and has been proven to be reliable and valid in our previous study [20]. The Cronbach’s α value for this inventory was 0.97 in the present study, which demonstrated excellent internal consistency reliability.

#### Burnout

Burnout was measured using the Chinese version of the Maslach Burnout Inventory General Survey (MBI-GS), which has been validated and proven to be reliable for use with Chinese nurses [35]. MBI-GS consists of three dimensions and 15 items: exhaustion (5 items), cynicism (4 items), and professional efficacy (6 items). Each item is scored on a 7-point Likert scale ranging from 0 (never) to 6 (every day). The Cronbach’s α for MBI-GS in this study was 0.82, suggesting that this scale had good internal consistency reliability. The total score of burnout was the weighting sum of the three dimensions: 0.40 × average score of exhaustion + 0.30 × average score of cynicism + 0.40 × average score of professional efficacy [35]. According to the cutoff proposed by Kalimo, the total score of MBI-GS was classified into three levels: 0 − 1.49, no burnout; 1.50 − 3.49, moderate burnout; 3.50 − 6, severe burnout [36].

#### Control variables

Demographic, socioeconomic, and job characteristics were included as control variables according to previous reviews and studies [1, 5]. Demographic characteristics included sex, age, and marital status. Socioeconomic characteristics included education level and monthly income. The job characteristics included the department (internal medicine, surgery, obstetrics and gynecology, pediatrics, emergency, and other), the professional title (primary, intermediate, and senior), the employment status (formal or contract), the working years (< 5, ≥ 5), working hours per week (≤ 40, 41 − 50, 51 − 60, >60), and the night shift work per week (0, 1 − 2, ≥ 3).

### Statistical analysis

The statistical analysis followed the BMJ guideline [37]. Descriptive statistics were used to describe the basic characteristics of the respondents and the distribution of key study variables. Numbers and percentages were used to describe categorical variables, and means and standard deviations were used to describe continuous variables. Skewness, kurtosis, and normality of continuous variables were tested using the “sktest” and “ladder” commands in Stata. For continuous variables with normal distribution, independent sample t-tests and one-way analysis of variance (ANOVA) were used to compare differences in depressive symptoms between subgroups of category independent variables. For non-normal distributive continuous variables, the Kruskal-Wallis nonparametric rank test was used to compare differences between subgroups [37, 38]. We used Harman’s one-factor test to assess common method variance (CMV) and found that 3 eigenvalues exceeded 1 and the factor with the highest eigenvalue accounted for only 32% of the variance in the data. The results demonstrated that there was no significant CMV in this study according to the rule of thumb [39]. The Pearson correlation coefficient (*r*) was calculated to evaluate the bivariate relationships for all key study variables. The PROCESS macro for SPSS provided by Hayes was used to test the effects of mediation [40]. This approach was based on ordinary least squares regression and the bootstrap resampling method [41], which is considered to have greater statistical power than the causal step approach [42]. We used model 6 in PROCESS macro to estimate the mediation effect and performed 5000 bootstrap re-samples to calculate the 95% bias-corrected confidence intervals (BCI). In the model, WPV was included as a categorical independent variable, FFWV and burnout were two mediators, and depressive symptoms were the dependent variables. The core hypothesis model we tested was how WPV influenced depressive symptoms through FFWV and burnout. The total effects of WPV on depressive symptoms were decomposed into direct effect and indirect effect. The postulated direct and indirect effects were considered significant when the 95% BCI did not cover zero. Descriptive statistics and correlation analysis were performed using Stata 15.0, and mediation analysis was performed using SPSS 26.0.

## Results

### Basic characteristics of the respondents

Among the 1,893 nurses sampled, most were women (93.9%) and married (80.1%), with an average age of 33.9 ± 7.3 years. Most of the respondents had a bachelor’s degree (87.9%) and earned more than 5000 Chinese Yuan (CNY) per month (82.4%). Nurses in the departments of internal medicine and surgery accounted for a high proportion of the sample at 28.3% and 39.2%, respectively. Approximately half of the nurses had primary (47.3%) or intermediate (48.4%) professional titles, three-quarters were contract employees, and 81.7% had worked for more than five years. Among nurses, 91.2% worked more than 40 h and 96.5% had at least one night shift per week. There were significant differences (*P* < 0.05) in depressive symptoms in the subgroups of education, income, department, professional title, type of employment, working years, working hours per week, and night work shifts per week, but there were no significant differences between the subgroups of gender and marital status (Table 1).

**Table 1:** Characteristics of the sampled nurses in Shandong province, China in 2020 (*N* = 1893)

Variable	Category	*N* (%)	Depressive symptoms, means (SD)	*P*
Depressive symptoms score		1893 (100)	9.2 (5.4)	-
Depressive symptoms	No	1025 (54.1)	-	-
	Yes	868 (45.9)	-	-
Workplace violence	No	252 (13.3)	6.5 (4.5)	< 0.001
	Yes	1641 (86.7)	9.6 (5.4)	
Burnout	No	64 (3.4)	7.2 (4.7)	< 0.001
	Moderate	1567 (82.8)	8.5 (4.9)	
	Severe	262 (13.8)	14.0 (5.7)	
Fear of future workplace violence, means (SD)		67.4 (17.3)	-	-
Age, years, means (SD)		33.9 (7.3)	-	-
Gender	Male	116 (6.1)	8.5 (5.7)	0.13
	Female	1777 (93.9)	9.2 (5.4)	
Marital status	Single	377 (19.9)	9.1 (5.4)	0.81
	Married	1516 (80.1)	9.2 (5.4)	
Education background	College diploma or below	138 (7.3)	10.0 (5.6)	0.003
	Bachelor	1664 (87.9)	9.2 (5.3)	
	Master or above	91 (4.8)	7.6 (6.0)	
Monthly income, CNY	<5000	333 (17.6)	9.2 (5.7)	< 0.001
	5000—8000	851 (45.0)	9.7 (5.4)	
	>8000	709 (37.5)	8.6 (5.3)	
Department	Internal medicine	535 (28.3)	7.8 (4.9)	< 0.001
	Surgery	743 (39.2)	9.2 (5.6)	
	Obstetrics and gynecology	95 (5.0)	9.5 (5.4)	
	Pediatrics	135 (7.1)	9.4 (4.9)	
	Emergency	164 (8.7)	10.4 (5.6)	
	Other	221 (11.7)	8.5 (5.1)	
Professional title	Primary	896 (47.3)	9.4 (5.3)	< 0.001
	Intermediate	916 (48.4)	9.3 (5.5)	
	Senior	81 (4.3)	5.5 (4.8)	
Employment type	Formal	478 (25.3)	8.6 (6.1)	0.011
	Contract	1415 (74.7)	9.4 (5.2)	
Working years, years	<5	347 (18.3)	8.5 (5.3)	0.010
	≥ 5	1546 (81.7)	9.3 (5.4)	
Working hours per week, hours	≤ 40	166 (8.8)	9.0 (5.9)	< 0.001
	41—50	1289 (68.1)	8.9 (5.2)	
	51—60	260 (13.7)	9.8 (5.5)	
	>60	178 (9.4)	10.6 (5.9)	
Night work shift per week, days	0	67 (3.5)	6.3 (4.4)	< 0.001
	1—2	1660 (87.7)	9.2 (5.4)	
	≥ 3	166 (8.8)	10.3 (5.4)	

*Abbreviations* CNY, Chinese Yuan; SD, Standard Deviation

^1^USD≈6.96 CNY (August 2020 exchange rate)

### Prevalence of workplace violence and depressive symptoms among nurses

The prevalence of WPV among nurses was 86.7% and the majority exhibited moderate (82.7%) or severe (13.8%) burnout. The mean scores of FFWV and CESD-10 were 67.4 ± 17.3 and 9.2 ± 5.4, respectively. The CESD-10 scores for nurses with WPV experience were significantly higher than those without such experience (*P* < 0.001). Furthermore, nurses with severe burnout had higher CESD-10 scores than those with moderate or no burnout (*P* < 0.001) (see Table 1).

### Correlations between depressive symptoms and independent variables

As shown in Table 2, depressive symptoms were positively associated with WPV (*r* = 0.196, *P* < 0.001), burnout (*r* = 0.340, *P* < 0.001), and FFWV (*r* = 0.171, *P* < 0.001). WPV, burnout, and FFWV also had positive significant interrelationships (*P* < 0.001).

**Table 2:** Pearson’s correlation coefficients between depressive symptoms and workplace violence, burnout, and fear of future workplace violence among nurses in Shandong province, China in 2020 (*N* = 1893)

Variable	Depressive symptoms	Workplace violence	Burnout	Fear of future workplace violence
Depressive symptoms	1			
Workplace violence	0.196^a^	1		
Burnout	0.340^a^	0.141^a^	1	
Fear of future workplace violence	0.171^a^	0.169^a^	0.204^a^	1

^a^The correlation is significant at level of 0.001 (tow-tailed)

### Mediation effect of workplace violence on depressive symptoms through fear of future workplace violence and burnout

**Fig. 1:**
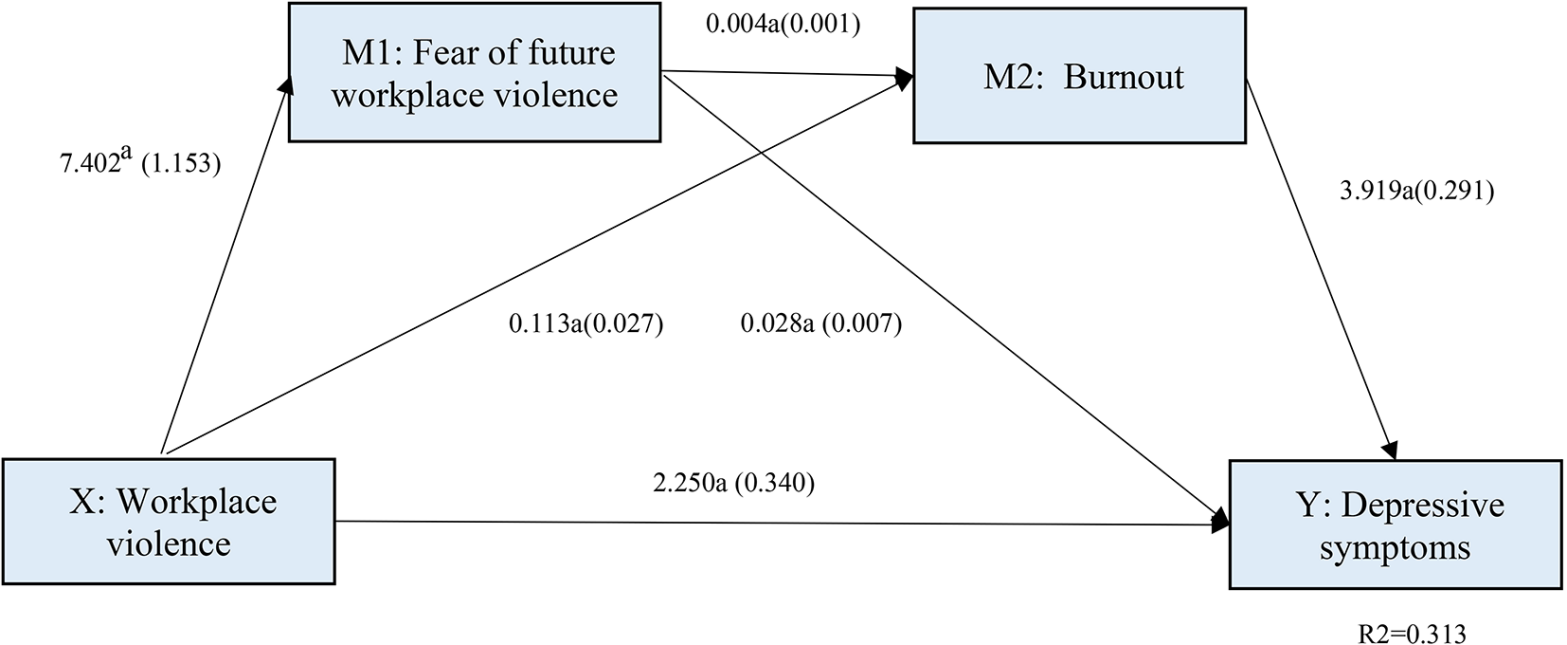
Serial multiple mediation of fear of future workplace violence and burnout in relationship between workplace violence and depressive symptoms among tertiary hospital nurses in Shandong province, China in 2020 (*N* = 1893). Non-standardized coefficients and standard error presented in the parentheses; ^a^*P* < 0.001

The results of the serial multiple mediation analysis are shown in Fig. 1; Table 3. Gender and marital status were not significantly correlated with depressive symptoms and therefore excluded from the final full regression models. First, as Fig. 1 shows, the regression results of the serial multiple mediation analysis confirmed a significant positive correlation (*P* < 0.001) between WPV and depressive symptoms and between FFWV and burnout, after adjusting for other control variables. The unstandardized coefficients of WPV and FFWV, burnout and depressive symptoms were 7.402, 0.113 and 2.250, respectively. Second, as seen in Table 3, the total effects (c = 3.019, *P* < 0.001) and the direct effect (c*’*=2.250, *P* < 0.001) of WPV on depressive symptoms were significant. Third, the indirect path through a single mediator (WPV→FFWV→depressive symptoms and WPV→burnout→depressive symptoms) and the indirect path through serial mediators (WPV→FFWV→burnout→depressive symptoms) were all statistically significant. The effects of the three indirect approaches were 0.203 (95% BCI: 0.090 to 0.348), 0.443 (95% BCI: 0.262 to 0.642) and 0.123 (95% BCI: 0.070 to 0.189), respectively. Finally, the total indirect effect (0.769, 95% BCI: 0.543 to 1.012) was also statistically positively significant. In the full model, the indirect effects mediated by FFWV and burnout accounted for approximately 25.5% of the total effects of WPV on depressive symptoms. Among the three indirect paths, path 2 (WPV→burnout→depressive symptoms) explained most of the indirect effects (57.6%), while path 1 (WPV→FFWV→depressive symptoms) accounted for 26.4% of indirect effects and path 3 (WPV→FFWV→burnout→depressive symptoms) only explained 16.0%.

**Table 3:** Total, direct, and indirect effects of workplace violence on depressive symptoms through fear of future workplace violence and burnout among nurses in Shandong province, China in 2020 (*N* = 1893)

Paths	Effects	Standard error	Bootstrap 95% confidence interval		*P*
Total effects	3.019	0.354	2.324	3.713	< 0.001
Direct effect	2.250	0.340	1.583	2.917	< 0.001
Total indirect effect	0.769	0.122	0.543	1.012	-
Indirect effect 1: X→M1→Y	0.203	0.066	0.090	0.348	-
Indirect effect 2: X→M2→Y	0.443	0.097	0.262	0.642	-
Indirect effect 3: X→M1→M2→Y	0.123	0.030	0.070	0.189	-

-, not applicable. X, workplace violence; M1, fear of future workplace violence; M2, burnout; Y, depressive symptoms

## Discussion

To our knowledge, this is the first study to explore the serial multiple mediation of FFWV and burnout in the relationship between WPV and depressive symptoms among nurses. The main findings of this study were: (1) there was a high prevalence of WPV and depressive symptoms among Chinese nurses; (2) WPV not only had direct negative effects on depressive symptoms, but also had indirect negative effects mediated by FFWV and burnout. This study provided valuable and precise evidence for policy makers and hospital managers to take effective measures to improve nurses’ mental health.

Our study showed that nurses with lower educational level, lower income, more night shift work per week, more years of work, longer work hours per week, or working in the emergency department were more likely to have depressive symptoms, which is consistent with previous studies [2, 5, 43, 44]. Our study also showed that contract nurses were more likely to experience depressive symptoms compare to the formal employee. Due to the inequitable benefit structures of nurses, contract nurses have a lower level of job satisfaction [45], which can cause them to experience depressive symptoms [46].

Our results revealed that the prevalence of depressive symptoms among Chinese nurses was 45.9%, which was higher than the rate recorded among American nurses (18%) [4] and Japanese nurses (38%) [47]. An explanation for this phenomenon is the high workloads. According to World Health Statistics 2023, the number of nurses per 10,000 people in China was significantly fewer than in the United States and Japan [48]. The heavy workloads can cause nurses to experience depressive symptoms [1]. Another reason is the high prevalence of WPV. Our findings showed a high incidence rate of WPV among Chinese nurses (86.7%), which was higher than the global prevalence of WPV against nurses (44.9%) [49]. Previous studies have found that WPV was associated with nurses’ depressive symptoms [50, 51]. Both the high prevalence of depressive symptoms could negatively affect the health status of nurses and the quality of nursing work they can perform. Therefore, it is essential that the level of depressive symptoms among Chinese nurses attract the attention of hospital managers.

In this study, we confirmed a significant association between WPV and depressive symptoms among nurses, which is consistent with the results of previous studies [50, 51]. An explanation is that, as a hindrance stressor, the pressure of insecurity at work is too difficult for nurses to overcome, which can cause emotional distress and hinder their career development, ultimately leading to deterioration of their mental health [14]. Another reason is that frequent WPV makes nurses feel disrespected in terms of their expertise and subsequently triggers the development of depressive symptoms [15]. Our data showed that nurses who had experienced WPV had much higher scores for depressive symptoms than those who did not (9.60 vs. 6.49). Therefore, exploring the underlying mechanisms between WPV and depressive symptoms could be very helpful in reducing the impact of WPV on depressive symptoms.

Our findings showed that WPV has a direct effect and indirect effect on depressive symptoms among Chinese nurses. We found that FFWV and burnout were two mediators between WPV and depressive symptoms and that WPV can affect depressive symptoms in three ways. First, WPV can affect depressive symptoms through FFWV. Nurses who experienced WPV were more likely to have high levels of FFWV, which in turn could lead to depressive symptoms. Research has shown that previous WPV experiences can generate FFWV in victims [20, 21]. Furthermore, Yao found that FFWV can affect the self-image and confidence of individuals, make them feel unsafe, and ultimately negatively affect their mental health [52]. Second, WPV can affect depressive symptoms through burnout. Nurses who experienced WPV were more likely to have high levels of burnout, which could directly lead to more depressive symptoms. Liu reported that WPV was associated with a higher incidence of burnout among nurses [53]. Furthermore, a study in Brazil found that nurses with burnout are more likely to develop depressive symptoms [26]. Third, the results of the serial multiple mediator model showed that the WPV experience was first associated with high levels of FFWV, followed by increased burnout, which in turn led to more depressive symptoms. Previous studies have also reported that prior experience with WPV is associated with high levels of FFWV among nurses [21]. Furthermore, Portoghese found that FFWV is associated with burnout among medical personnel [22], while Rogers found that FFWV is an important antecedent of burnout among employees [28]. Therefore, people with high levels of FFWV are more likely to experience higher levels of burnout, which could lead nurses to develop more depressive symptoms [26]. By examining the underlying mechanisms between WPV and depressive symptoms among nurses, our results suggested that reducing the incidence of WPV, reducing FFWV, and reducing burnout were all possible interventions that could be implemented to reduce the appearance of depressive symptoms among nurses.

### Strengths and limitations

This study showed that there was a high prevalence of WPV and depressive symptoms among Chinese nurses and found a serial multiple mediation of FFWV and burnout in the relationship between WPV and depressive symptoms among nurses, which fills the literature gap. These findings contribute to the development of effective interventions that aim to improve the mental health and well-being of nurses and reduce their WPV, FFWV, and burnout. Furthermore, this study had a large sample size and a high response rate. There are several limitations to this study. First, since this was a cross-sectional study, we could not examine the causal relationships between WPV, FFWV, burnout, and depressive symptoms. Second, the data in this survey were collected based on the nurses’ self-reports; therefore, there may be inaccurate responses, causing bias. Third, the participants in this study were only from tertiary hospitals in Shandong province, which could limit the generalizability of the findings to nurses from primary and secondary hospitals or other provinces in China. Fourth, although the present study was conducted in the context of China’s progress in containing COVID-19 and the gradual relaxation of restrictions, it is still necessary to consider the impact of the COVID-19 epidemic on the mental health of nurses.

### Implications

Based on the findings of this study, we suggest that health policy makers and hospital managers should: (1) pay more attention to the nurses’ mental health; (2) take effective measures to reduce the incidence of WPV, such as implementing policies and legislation to protect the safety of health care workers, providing nurses with appropriate training programs to prevent and stop WPV [54], establishing an effective WPV reporting system and encouraging nurses to participate in reporting [55]; (3) conduct the necessary psychological counseling programs to reduce nurses’ FFWV; and (4) manage the nurses’ work hours and workloads of nurses more reasonably [41], guarantee vacation time, and reduce the negative effects of burnout on depressive symptoms.

## Conclusions

This study found a high incidence of WPV and depressive symptoms among Chinese nurses. WPV had direct and indirect effects on depressive symptoms. Furthermore, both FFWV and burnout mediated the relationship between WPV and depressive symptoms. Implementing targeted interventions geared toward the specific reduction of WPV, FFWV, and burnout is essential to reduce the prevalence of depressive symptoms and improve the mental health of nurses.

## Data Availability

The datasets generated and/or analysed during the current study are not publicly available due to agreements with participants who restricted data sharing but are available from the corresponding author on reasonable request.

## Abbreviations

BCI: Bootstrap confidence interval
CESD: Center for Epidemiologic Studies-Depression
CMV: Common method variance
CNY: Chinese Yuan
FFWV: Fear of future workplace violence
MBI-GS: Maslach Burnout Inventory-General Survey
SD: Standard deviation
WHO: World Health Organization
WPV: Workplace violence
